# Substantially elevated serum glutamate and CSF GOT-1 levels associated with cerebral ischemia and poor neurological outcomes in subarachnoid hemorrhage patients

**DOI:** 10.1038/s41598-023-32302-3

**Published:** 2023-03-31

**Authors:** Silvia Snider, Luigi Albano, Filippo Gagliardi, Stefano Comai, Francesca Roncelli, Pierfrancesco De Domenico, Edoardo Pompeo, Pietro Panni, Nicole Bens, Maria Rosa Calvi, Pietro Mortini, Angela Ruban

**Affiliations:** 1 Department of Neurosurgery and Gamma Knife Radiosurgery, IRCCS San Raffaele Scientific Institute, Milan, Italy; 2 Department of Pharmaceutical and Pharmacological Sciences, University of Padua, Padua, Italy; 3 Department of Neuroradiology, IRCCS San Raffaele Scientific Institute, Vita-Salute University, Milan, Italy; 4 Behavioral Neuroscience, Human Movement Science, Mathematics, Pre-Medicine, Northeastern University COS, Boston, MA USA; 5 Department of Neurocritical Care, IRCCS San Raffaele Scientific Institute, Vita-Salute University, Milan, Italy; 6 Sackler Faculty of Medicine, Steyer School of Health Professions, Tel Aviv University, P.O. Box 39040, 6997801 Tel-Aviv, Israel; 7 Sagol School of Neuroscience, Tel-Aviv University, P.O. Box 39040, 6997801 Tel-Aviv, Israel

**Keywords:** Neuroscience, Neurology

## Abstract

Brain injury and cerebral vasospasm during the 14 days after the subarachnoid hemorrhage (SAH) are considered the leading causes of poor outcomes. The primary injury induces a cascade of events, including increased intracranial pressure, cerebral vasospasm and ischemia, glutamate excitotoxicity, and neuronal cell death. The objective of this study was to monitor the time course of glutamate, and associated enzymes, such as glutamate–oxaloacetate transaminase (GOT1), glutamate-pyruvate transaminase (GPT) in cerebrospinal fluid (CSF) and serum, shortly after SAH, and to assess their prognostic value. A total of 74 participants participated in this study: 45 participants with SAH and 29 controls. Serum and CSF were sampled up to 14 days after SAH. SAH participants' clinical and neurological status were assessed at hospitalization, at discharge from the hospital, and 3 months after SAH. Furthermore, a logistic regression analysis was carried out to evaluate the ability of GOT1 and glutamate levels to predict neurological outcomes. Our results demonstrated consistently elevated serum and CSF glutamate levels after SAH. Furthermore, serum glutamate level was significantly higher in patients with cerebral ischemia and poor neurological outcome. CSF GOT1 was significantly higher in patients with uncontrolled intracranial hypertension and cerebral ischemia post-SAH, and independently predicted poor neurological outcomes.

## Introduction

Despite the considerable efforts of neuro-clinicians worldwide, subarachnoid hemorrhage (SAH) remains a life-threatening condition^1^. Early brain injury and cerebral vasospasm during the 14 days after the injury are considered the main causes of poor outcome in patients with SAH^2–4^. The primary injury induces a cascade of events, including increased intracranial pressure (ICP), blood–brain barrier (BBB) dysfunction, glutamate excitotoxicity, and neuronal cell death^5–7^.

Glutamate excitotoxicity is involved in early damage and neuronal death in neurotrauma^8,9^. It has also been implicated in the formation of cerebral edema, which is an independent risk factor for poor outcomes after SAH^10,11^. Monitoring the extracellular cerebrospinal fluid (CSF) glutamate concentration has been proposed as a potential tool for predicting poor outcome following SAH^12,13^.

Under the pathological conditions associated with a large increase of extracellular glutamate in the brain, glutamate has been reported to diffuse from the extracellular space into the bloodstream following a concentration gradient, mainly via glutamate/glutamine transporters located on the brain endothelium capillaries^14,15^. The efflux of glutamate from the brain to the blood could explain the elevated serum glutamate levels observed in brain pathologies^16–18^. Glutamate is regulated in the brain and blood by two closely linked systems and their mutual influence plays an important role in the brain pathophysiology^14,19,20^. GOT1 plays a critical role in regulating glutamate levels and reducing excess extracellular glutamate levels by catalyzing the reversible transformation of oxaloacetate and glutamate to aspartate and α-ketoglutarate. Therefore, high GOT1 activity can reduce excitotoxicity after neurotrauma. However, serum glutamate levels have not been tested as a possible marker for monitoring patients' neurological status during their hospital stay or as a potential prognostic tool for the post-SAH neurological outcome. Serum glutamate level is predominantly regulated by two enzymes, GOT1 and GPT^21^. We and our colleagues previously reported a neuroprotective effect of reducing blood glutamate levels by the administration of recombinant GOT1 and oxaloacetate in SAH, spinal cord injury, stroke, and Paraoxon intoxication animal models^22–25^. Although a few studies have suggested a possible neuroprotective effect of brain GOT1 in various neuro-pathologies, including stroke, the role of brain GOT1 activity in SAH has yet to be studied^26,27^.

Given the above, the aim of the present study is to investigate the time course of glutamate, aspartate (one of the products of glutamate transamination), GOT1, and GPT in CSF and serum, during the first two weeks after aneurism rupture, their correlation with neurological status at admission, and the possible prognostic value in SAH patients.

## Materials and methods

### Ethics approval and consent to participate

The study was carried out at the San Raffaele Medical Center, Milano, Italy, was conducted according to the principles delineated in the Declaration of Helsinki, and was approved by the San Raffaele Ethics Committee (approval number NCH01-2016). All methods were performed in accordance with the San Raffaele Medical Center guidelines and regulations. The research participants were included after written and oral informed consent was obtained either from the patients or from one of their family members in case of inability to give consent.

### The research population, inclusion and exclusion criteria

The research group consisted of forty-five patients, in addition to two control groups (14 patients with benign pituitary pathology and 15 healthy subjects).

#### Research group

**Figure 1:**
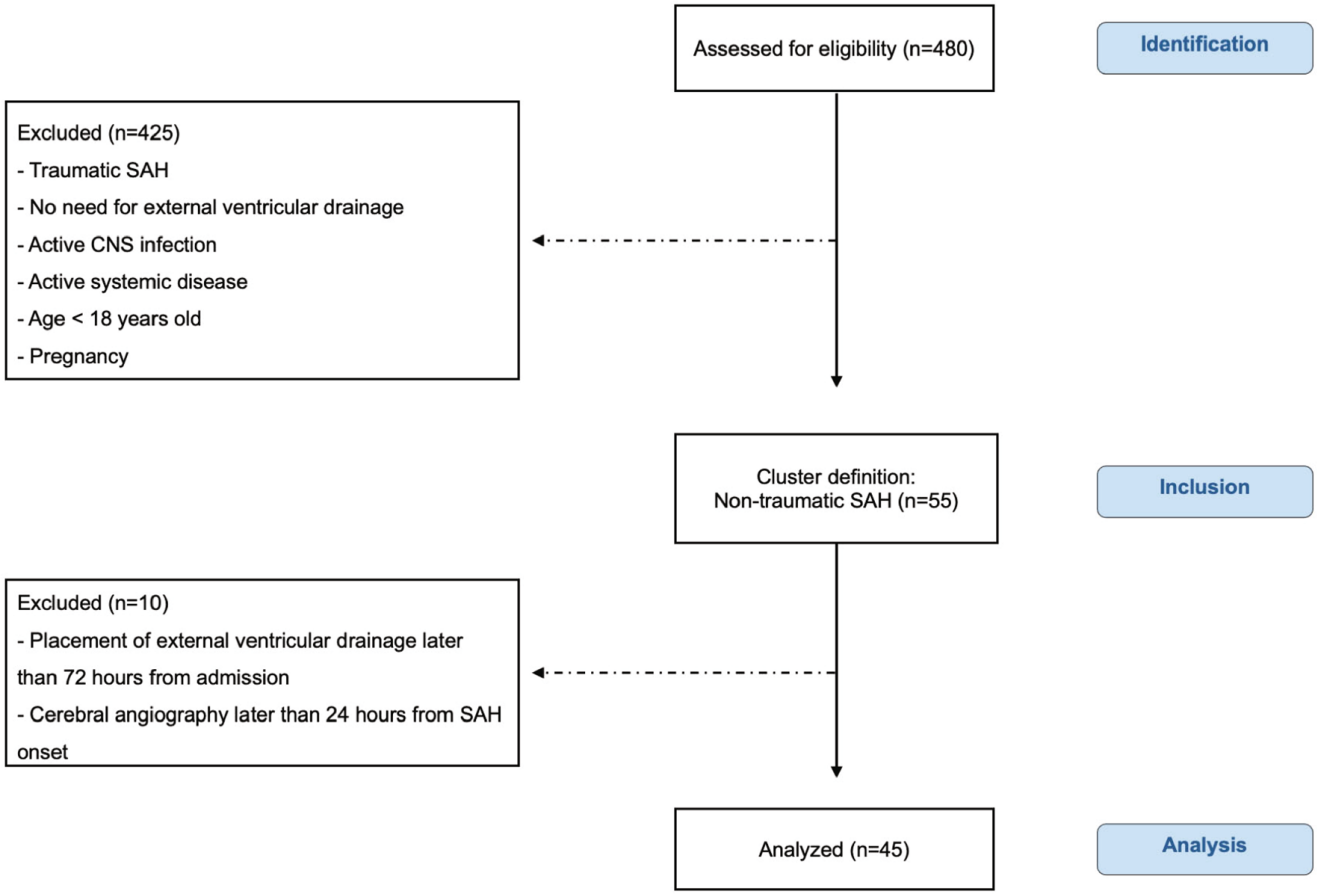
Flowchart illustrating the inclusion/exclusion criteria of individuals in the study. Chart data for 480 patients who were admitted for spontaneous SAH to the Intensive Care Unit in the Neurosurgical Department, IRCCS Ospedale San Raffaele, Milan, Italy. According to inclusion and exclusion criteria, 45 patients affected by spontaneous non-traumatic SAH were included in the study. Abbreviations: CNS = Central nervous system; SAH = Subarachnoid hemorrhage; According to inclusion and exclusion criteria, 45 patients affected by spontaneous non-traumatic SAH were included in the study. Abbreviations: CNS = Central nervous system; SAH = Subarachnoid hemorrhage.

Forty-five patients with spontaneous SAH were referred to the Intensive Care Unit in the Neurosurgical Department during the study recruitment period, from October 2016 to October 2021 (Fig. 1). The inclusion criteria were: (1) non-traumatic SAH diagnosed by non-contrast computed tomography (CT); (2) insertion of external ventricular drainage (EVD) within 72 h post-admission; and (3) cerebral angiography within the first 24 h. The exclusion criteria for the study were: (1) traumatic SAH; (2) no need for external ventricular drainage; (3) active CNS infection; (4) active systemic disease (e.g., malignancy, cirrhosis, or renal failure); (5) age below 18; and (6) pregnancy.

#### Control groups

Fifteen healthy volunteers were enrolled in a control group for the serum glutamate and enzyme analyses. In addition, 14 patients affected by benign pituitary pathology were enrolled in a control group for CSF analysis. These patients were candidates for trans-sphenoidal (TNS) surgery, which involves CSF drainage as part of the scheduled procedure. As for the SAH group, CSF samples were collected at the end of the surgery, at the same time as blood samples.

### Management, definitions, and end points

At admission, clinical status was assessed by means of the Glasgow Coma Scale (GCS) and Hunt Hess. In addition, the Fisher scale grade was determined based on the initial CT scan. At admission, patients were divided into two categories: a good grade (GCS ≥ 9, HH I–II, Fisher I–III) and a poor grade (GCS ≤ 8, HH III–V, Fisher IV). Treatment for SAH was according to the best clinical practice. In case of a diagnosis of cerebral vascular aneurysm at angiography, the aneurism was isolated from the normal circulation either by surgical clipping or coiling, depending on the morphological angiographic characteristics of the aneurism. The presence of vasospasm was assessed using angiography, which was performed in case of exacerbation of clinical and/or vital signs of the patients during hospitalization. A control CT scan is performed on 5–7 days after SAH, discovered the presence of hypodense areas due to ischemia. Demographic and clinical variables, including age, gender, symptoms at onset, comorbidity, and treatment modality (clipping or coiling) were recorded. Thirty-eight patients underwent invasive treatment of the underlying aneurism, consisting of either early clipping (n = 9) or coiling (n = 28) or both (n = 1). Seven patients had a negative angiography for cerebrovascular abnormalities. All patients had elevated intracranial pressure at admission, while persistently elevated intracranial pressure (ICP > 20 mmHg) resistant to medical treatment was present in 13 patients. It is a relatively common complication of SAH, especially in patients with a poor neurological condition at admission^28^. Patients presenting with SAH are admitted to an intensive care unit for hemodynamic and neurological monitoring, and commonly an external intraventricular catheter (EVD) is inserted for drainage of CSF and recording intracranial pressure. This is followed by definitive treatment of the ruptured aneurysm or pathology causing the SAH^29^. In this study, all SAH participants underwent surgery for placement of an external ventricular drain (EVD) as part of the treatment protocol in an intensive care unit. The presence of neurological deficits was assessed by an independent neurosurgeon at discharge from the hospital and outcome was assessed 3 months after SAH according to the Glasgow Outcome Scale (GOS). The patients were then stratified into good (GOS IV–V) versus poor (GOS I–III) outcome.

### Serum and CSF sample collection

At admission, blood samples were collected for GOT1, GPT, glutamate, and aspartate level measurements. CSF samples were collected from the EVD at three different time points (day 0–3, 5–7, and 14 post-SAH). Venous blood and CSF samples were collected at the same time points. Control CSF samples were obtained intraoperatively under general anesthesia from age- and sex-matched patients who were candidates for trans-sphenoidal (TNS) surgery (n = 14). This procedure involved lumbar drainage placement as part of the treatment protocol. Blood samples from healthy volunteers (n = 15) were collected to analyze the serum GOT1, GPT, glutamate, and aspartate levels. All samples were immediately centrifuged, and supernatants were stored at − 20 °C until further assessment.

### Determination of free glutamic acid and aspartic acid in CSF

The levels of the glutamate and aspartate were evaluated by high performance liquid chromatography (HPLC), as previously described^30^. This analysis was based on o-phtaldialdehyde (OPA) pre-column amino acid derivatization and was carried out on a reversed–phase C18 column using an isocratic run and fluorometric detection ^31^.

### Determination of GOT and GPT enzyme activity in CSF

GOT1 and GPT levels were measured using a Reflotron Plus, Sprint system (Roche), and test strips for GOT1 and GPT (10,745,120 and 10,745,123; Roche).

### Statistical analysis

Comparisons of the glutamate and GOT1 levels between the SAH participants and the control groups are presented as mean ± standard deviation (SD). The magnitude of the differences in the levels of GOT1 and glutamate between the SAH and control groups was assessed using an effect size test. To compare between a good or poor outcome, the chi-square test was used for categorical variables, and Student’s *t-test* for continuous variables. The comparison between glutamate in serum and GOT1 in CSF samples was determined by Mann Whitney test. Pearson’s analyses for bivariate correlations were used to compare CSF GOT1 and glutamate and serum glutamate levels. Finally, logistic regression analysis was used to evaluate the ability of GOT1 and glutamate levels to predict the neurological outcome. SPSS software version 25 was used for all statistical analyses.

### Data sharing

The data that support the findings of this study are available on request from the corresponding author. The data are not publicly available due to privacy or ethical restrictions.

## Results

### Characteristics of the participants

The flowchart of the study, including inclusion and exclusion criteria, is shown in Fig. 1. Flowchart illustrating the inclusion/exclusion criteria of individuals in the study. Chart data for 480 patients who were admitted for spontaneous SAH to the Intensive Care Unit in the Neurosurgical Department, IRCCS Ospedale San Raffaele, Milan, Italy. According to inclusion and exclusion criteria, 45 patients affected by spontaneous non-traumatic SAH were included in the study.

#### Research group

Participants were forty-five patients with spontaneous SAH requiring an external ventricular CSF drainage (EVD) to relieve intracranial pressure. Table 1 presents the main characteristics of the overall population, stratified by the outcome. The median age was 60 (range 33–92); 31 were females (69%). Overall, 40% of the research group presented with a Fisher grade of I–III and 69% with a Glasgow Coma Scale (GCS) value above 9. Thirty-eight (84%) participants presented radiological evidence of cerebral aneurism. The presence of an angiographically documented vasospasm and/or the presence of delayed hypodense area on CT scan was found in 16 patients. An equal number of participants were stratified as having a good or poor outcome according to the Glasgow Outcome Scale (GOS). The median hospital length of stay (LOS) was 31.5 days (range 16–157) and was significantly shorter in SAH patients with a good prognosis. Five participants (11%) died while in the hospital; four patients due to refractory intracranial hypertension and one patient as a result of systemic complications.

**Table 1:** Summary of characteristics of the 45 patients with SAH.

Variable	Good outcome GOS IV–V (n = 24)	Poor outcome GOS I–III (n = 21)	All N = 45	*p* value
Age, median, range	61 (33–92)	57.7 (42–79)	60 (33–92)	0.47
Gender, female	17 (38%)	14 (31%)	31 (69%)	0.77
Absence of aneurysm	6 (13%)	1 (2%)	7 (16%)	< 0.001
Ruptured aneurysm	18 (40%)	20 (44%)	38 (84%)	
HH I–II	14 (31%)	2(4%)	16 (35%)	< 0.001
Fisher grade I–III, n (%)	13 (29%)	5 (11%)	18 (40%)	< 0.001
GCS > 9	21 (47%)	10 (22%)	31 (69%)	< 0.001
Treatment, coiling	13(29%)	15(33%)	28 (62%)	
Controlled ICP (ICP ≤ 20 mm Hg)	22 (49%)	10 (22%)	25 (71%)	< 0.001
Absence of neurological deficit	17 (38%)	0	17 (38%)	< 0.001
LOS ≤ 30 days	18 (40%)	2 (4%)	20 (44%)	< 0.001

GOS, Glasgow Outcome scale at 3 months; HH, Hunt Hess scale; GCS, Glasgow Coma Scale; ICP, intracranial pressure; LOS, hospital length of stay.

#### Control groups

Since the collection of a CSF sample represents an invasive procedure, it is unethical to recruit healthy volunteers. Instead, patients who were candidates for trans-sphenoidal (TNS) surgery as CSF controls were enrolled since CSF drainage is part of the scheduled surgical procedure. The patients (n = 14) were in-patients in the Neurosurgery Department during the same period as the research group (Supplementary Table 1). As for the SAH group, CSF and blood were both collected at the end of the surgery. The serum markers (Supplementary Table 2) were also analyzed in blood samples from healthy volunteers (n = 15).

### GOT, GPT activity and glutamate and aspartate levels in CSF and serum

GOT1 activity in the serum of SAH participants did not differ significantly from those of the healthy and TNS control groups (SAH participants GOT1 34.3 ± 5.4 U/L versus 31.54 ± 6.5 U/L and 28.9 ± 6.9 U/L in the healthy and TNS groups, respectively).

**Figure 2:**
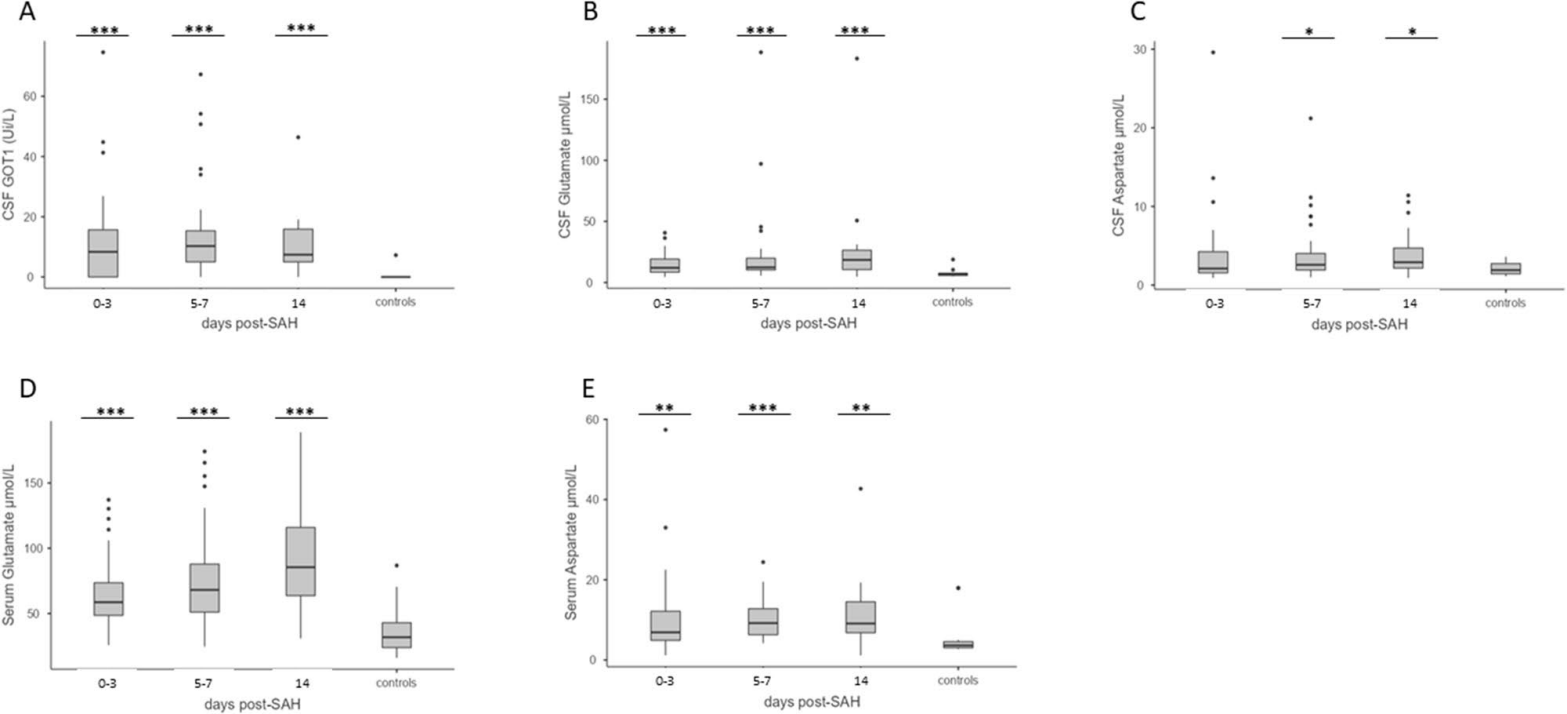
Levels of GOT1, glutamate and aspartate in CSF and serum. Elevated CSF GOT1 level (U/L) on days 0–3, 5–7, and 14 post-SAH versus CSF control samples, obtained from patients affected by benign pituitary pathology (**A**). Elevated CSF glutamate (**B**) and aspartate (**C**) levels on days 0–3, 5–7, and 14 post-SAH versus CSF control samples obtained from patients affected by benign pituitary pathology Data are shown as mean ± SD. Statistical analysis was performed using the two-tailed Student’s *t*-test. ***p < 0.001.. Elevated serum glutamate (**D**) and aspartate (**E**) levels on days 0–3, 5–7, and 14 post-SAH versus blood control samples of healthy volunteers. Data are shown as mean ± SD. Statistical analysis was performed using the Mann Whitney test. *p < 0.05, **p < 0.01, ***p < 0.001.

CSF GOT1 activity and glutamate levels, serum glutamate and aspartate levels were significantly elevated from day one (Fig. 2). The activity of CSF GOT1 was elevated in SAH participants at all time points but was undetectable in the TNS control group (˂ 5U/L) (SAH participants: days 0–3: 12.08 ± 1 5.54 U/LU/L; days 5–7: 13.9 ± 15.72U/L; day 14: 10.42 ± 10.61 U/L; controls: 2.83 ± 1.29 U/L; *p*-value ≤ 0.001; *p* ≤ 0.001; *p* ≤ 0.001, respectively), with a large size effect at all time points (days 0–3: *d* = 0.87; days 5–7: *d* = 0.99; and day 14: *d* = 1.19 respectively, Fig. 2A). Nevertheless, the GPT activity was undetectable in the CSF of both SAH participants and TNS controls. Also, the CSF glutamate level significantly increased after SAH on days 0–3, 5–7, and 14 (day 0–3: 14.25 ± 8.41 μM, days 5–7: 21.59 ± 31.23 μM; day 14: 25.68 ± 36.04 μM; *p*-value = 0.001; ≤ 0.001; and ≤ 0.001, respectively, TNS controls 7.62 ± 3.65 μM), with medium size effects (days 0–3: *d* = 0.62; days 5–7: *d* = 0.51; and day 14: *d* = 0.62 respectively, Fig. 2B). In addition, the aspartate level in the CSF was significantly elevated at 5–7 and 14 days post-SAH (day 0–3: 3.95 ± 5.25 μM, days 5–7: 3.94 ± 3.85 μM; day 14: 3.97 ± 2.95 μM; *p*-value ns; = 0.045; and = 0.023, respectively, TNS controls 2.09 ± 0.86 μM), with small and medium size effects (days 0–3: *d* = 0.42; days 5–7: *d* = 0.56; and day 14: *d* = 0.79, respectively (Fig. 2C).

Compared to the healthy controls, a significant elevation in the serum glutamate level was measured during the first two weeks after SAH (SAH participants: days 0–3: 66.08 ± 27.37 μM; days 5–7: 77.38 ± 37.04 μM; and day 14: 91.66 ± 42.66 μM; controls: 37.62 ± 19.94 μM; *p*-value ˂ 0.001), with a large size effect at all time points (days 0–3: *d* = 1.09; days 5–7: *d* = 1.17 and day 14: *d* = 1.45 respectively, Fig. 2D).

In serum aspartate, levels were significantly higher in the SAH participants at all time points (SAH participants: days 0–3: 10.41 ± 10.61 μM; days 5–7: 9.99 ± 4.89 μM; and day 14: 11.16 ± 8.29 μM; controls: 5.64 ± 5.26 μM; *p*-value = 0.004; ˂ 0.001; < 0.001), with medium and large size effects (days 0–3: *d* = 0.51; days 5–7: *d* = 0.87; and day 14: *d* = 0.75, respectively (Fig. 2E).

### Neurological diagnostic value of blood and CSF markers

**Figure 3:**
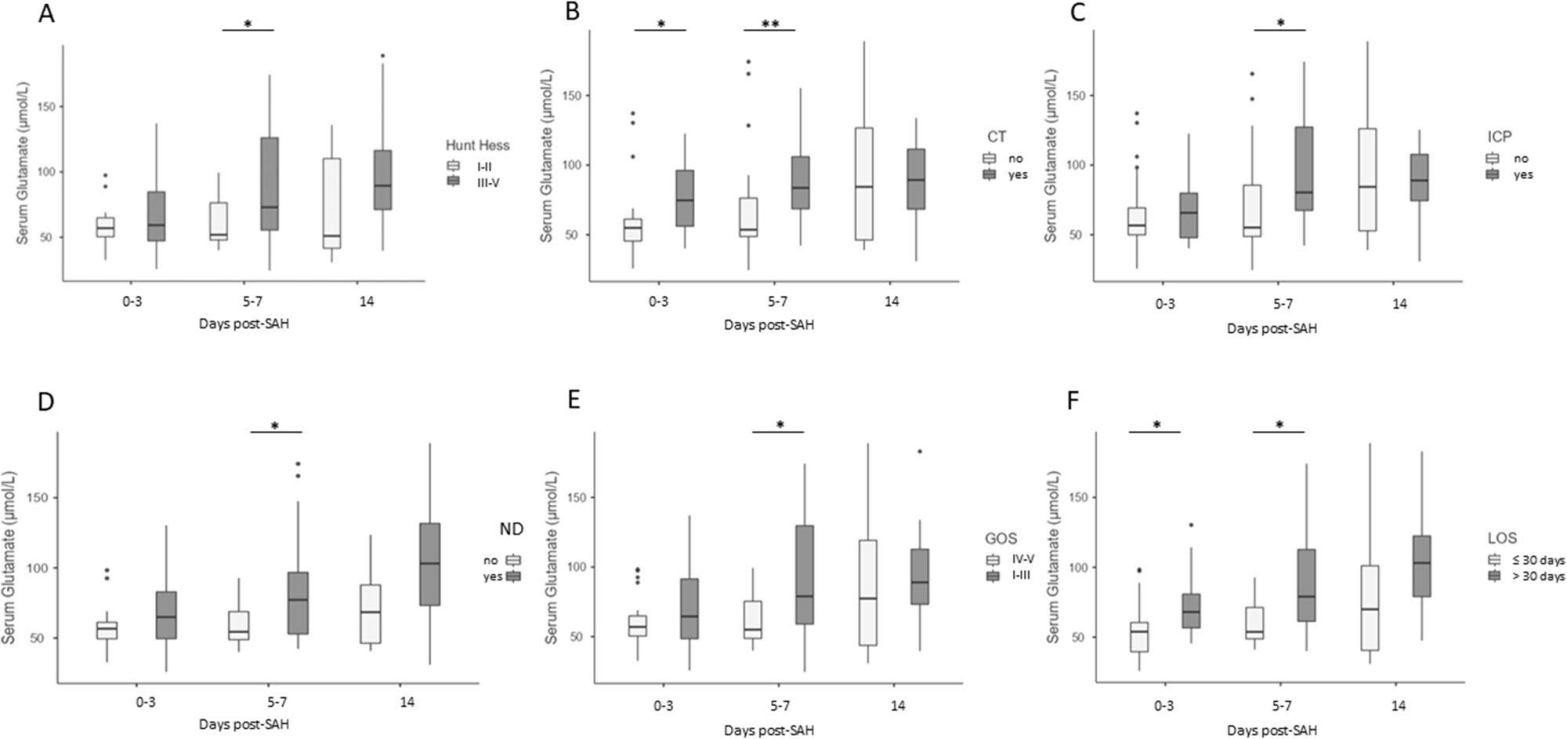
Diagnostic and Prognostic value of serum and CSF markers*.* High serum glutamate levels at days 5–7 correlated with higher HH (**A**). Serum glutamate levels at days 0–3, 5–7, and 14 post-SAH correlated with the presence of ischemia (hypodense lesions) on a brain CT scan (**B**) and increased intracranial pressure (ICP) (**C**). (**D**-**F**) Correlation between serum glutamate levels at days 0–3, 5–7, and 14 post-SAH and presence of neurological deficits at discharge (**D**), clinical outcome defined as “good” (GOS IV–V) versus “poor” (GOS I–III) 3 months after SAH (**E**), and hospital length of stay (LOS) (**F**). Data are expressed as the median and interquartile range. Statistical analysis was performed using the Mann Whitney test, *p < 0.05, **p < 0.01.

When SAH participants were stratified by good or poor grade at admission, a higher serum glutamate level at 5–7 was found to correlate with a higher HH score (*p*-value ≤ 0.022 (Fig. 3A). No correlations of serum glutamate or aspartate levels were observed with GCS or Fisher scale scores at admission. No correlations were found between CSF glutamate, aspartate levels, or GOT1 activity with clinical status at admission.

### Prognostic value of blood and CSF markers

**Figure 4:**
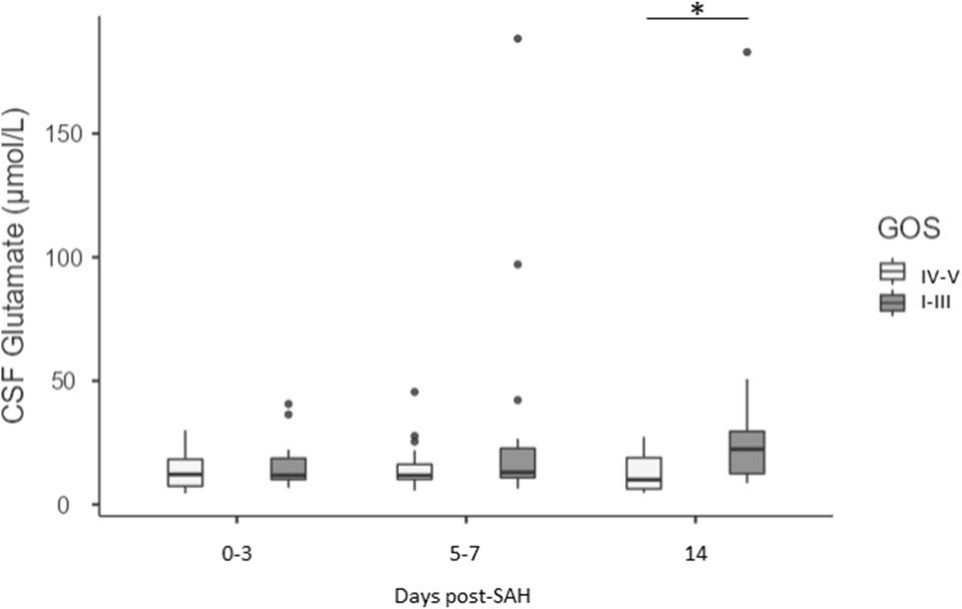
Correlations between CSF glutamate levels and clinical outcome Correlation between CSF glutamate levels at days 0–3, 5–7, and 14 post-SAH and outcome defined as “good” (GOS IV–V) versus “poor” (GOS I–III). Data are expressed as the median and interquartile range. Statistical analysis was performed using the Mann–Whitney test. *p < 0.05.

Significantly higher levels of serum glutamate at days 0–3 and 5–7 post-SAH was detected in patients with the presence of brain ischemia on the CT scan, and with high ICP at days 5–7 (*p*-value ≤ 0.03 and ≤ 0.031, respectively, Fig. 3B, C). Regarding the prognostic value of the serum glutamate, increased serum glutamate levels at days 0–3 and 5–7 post-SAH were associated with the presence of a neurological deficit at hospital discharge, with longer hospitalization period (LOS) and poor GOS at 3 months post-SAH, and the (*p*-value ≤ 0.033 and 0.009; *p*-value ≤ 0.018 and ≤ 0.014, respectively, Fig. 3D–F). CSF glutamate levels were found to be significantly higher in patients with poor GOS at 14 days post-SAH (*p*-value ≤ 0.047, Fig. 4).

**Figure 5:**
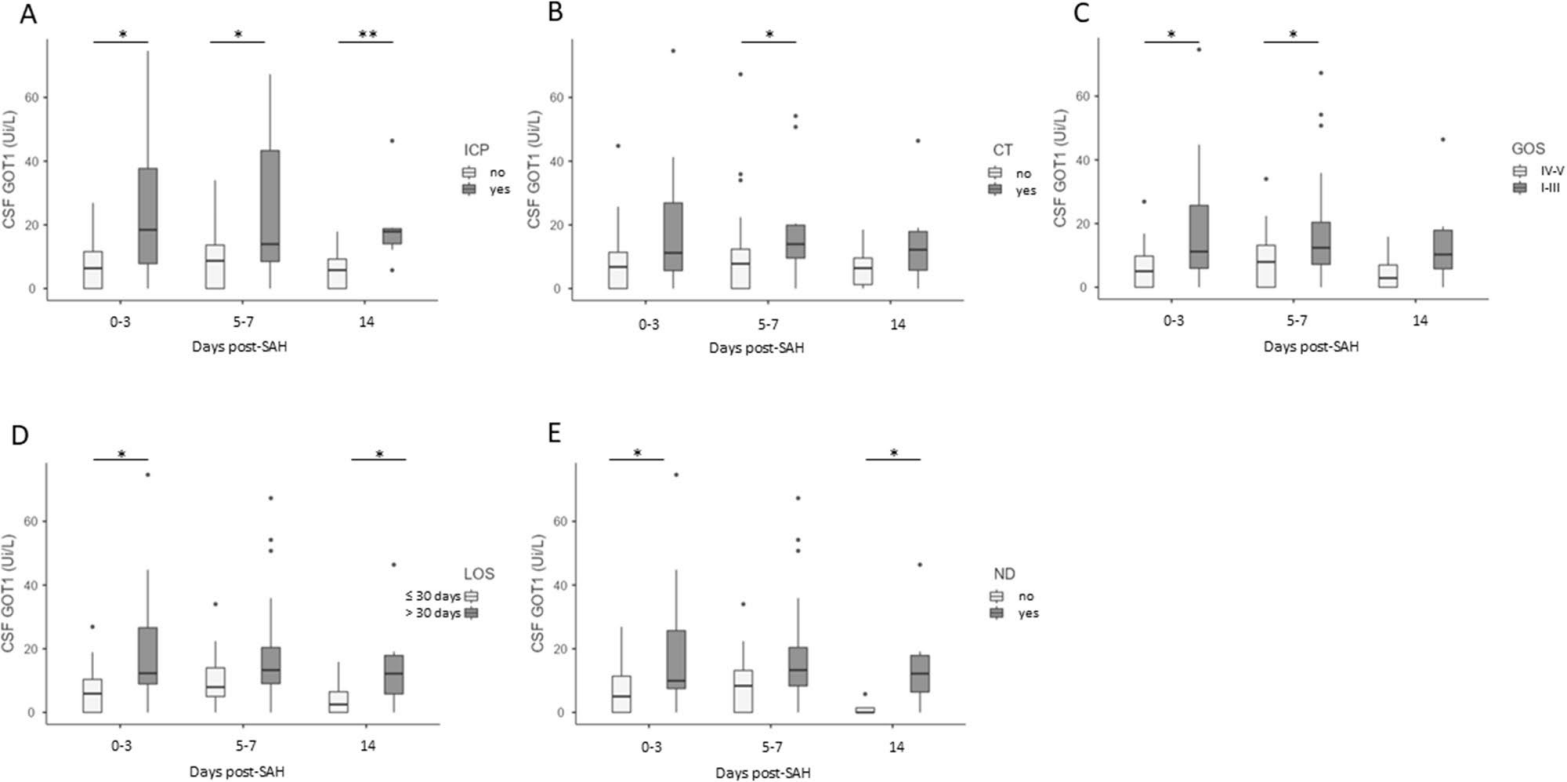
Correlations between CSF GOT1 levels and high ICP, brain ischemia, and clinical outcome. CSF GOT1 levels on days 0–3, 5–7, and 14 post-SAH, in association with the presence of increased intracranial pressure (ICP) (**A**), and at days 5–7 post-SAH with the presence of brain ischemia (hypodense lesion on a brain CT) (**B**). Correlation between CSF GOT1 levels at days 0–3, 5–7, and 14 post-SAH and outcome defined as “good” (GOS IV–V) versus “poor” (GOS I–III) 3 months post-SAH (**C**), hospital length of stay (LOS) (**D**), and the presence of neurological deficits at discharge (**E**). Data are expressed as the median and interquartile range. Statistical analysis was performed using the Mann–Whitney test (**B**), *p < 0.05, **p < 0.01.

Overall, in SAH participants higher activity of CSF GOT1 was detected in patients with increased ICP at all time-points and with the presence of brain ischemia on a CT scan at 5–7 days (ICP *p*-value ≤ 0.022; ≤ 0.05; and ≤ 0.005; CT *p*-value ≤ 0.03, respectively, Fig. 5A, B). No differences in CSF glutamate levels between patients with uncontrolled versus controlled ICP was found. The activity of CSF GOT1 at days 0–3 and 5–7 was higher in patients with unfavorable GOS 3 months later (*p*-value = 0.022; and 0.05, respectively) (Fig. 5C). When SAH participants were stratified by the presence of severe neurological deficit (ND), and length of hospitalization (LOS), significantly higher CSF GOT1 activity was measured at 0–3 and 14 days post-SAH in patients with prolonged LOS and severe ND (LOS more than 30 days, *p*-value ≤ 0.019 and 0.05; presence of neurological deficit *p*-value ≤ 0.05 and 0.018; respectively) (Fig. 5D, E).

Out of 32 patients with controlled ICP, twenty-two (69%) patients had a good neurologic outcome 3 months later (Table 1). A logistic regression analysis was utilized to determine whether serum or CSF glutamate levels and GOT1 activity at different time points represent an independent prognostic biomarker for ICP and poor neurological outcome. The analysis revealed a significant association between the activity of GOT1 in the CSF at days 0–3 and 5–7, and increased ICP (OR:1.097, *p* = 0.05; OR 1.072, *p* = 0.047; Table 2). In addition, a significant association was observed between serum glutamate levels and ICP at 5–7 days post-SAH (OR: 1.019, *p* = 0.05). Serum glutamate at day 5–7 was weakly but significantly associated with presence of neurological deficits (OR: 1.039, *p* = 0.043, Table 3) and poor prognosis (OR: 1029, *p* = 0.019, Table 4).

**Table 2:** Odds ratio (OR) of increased intracranial pressure (ICP) in 45 SAH patients during their hospitalization. Age, gender, and CSF GOT1 levels and serum glutamate as predictors.

Independent variables	OR	CI 95%	*p* value
Age	0.987	0.935–1.042	0.635
Gender	0.978	0.242–3.945	
CSF GOT1 0–3	1.097	1.001–1.207	**0.050**
CSF GOT1 5–7	1.072	1.001–1.148	**0.047**
CSF GOT1 14	1.251	0.964–1.622	0.092
Serum GLU 0–3	1.008	0.984–1.033	0.511
Serum GLU 5–7	1.019	1.001–1.039	**0.050**
Serum GLU 14	0.996	0.976–1.017	0.719

OR, odds ratio; CI, confidence interval; CSF, cerebrospinal fluid, GOT1, glutamate–oxaloacetate transaminase.

Significant values are in [bold].

**Table 3:** Odds ratio (OR) of the presence of neurological deficits in 45 SAH patients at hospital discharge. Age, gender, and serum glutamate levels as predictors.

Independent variables	OR	95% CI	*P* value
Age	1.032	0.977–1.091	0.255
Gender	0.802	0.211–3.043	
Serum GLU 0–3	1.023	0.991–1.056	0.163
Serum GLU 5–7	1.039	1.001–1.079	**0.043**
Serum GLU 14	1.021	0.996–1.047	0.107

OR, odds ratio; CI, confidence interval; SGLU, serum glutamate.

Significant values are in [bold].

**Table 4:** Odds ratio (OR) of poor outcome at 3 months. Age, gender and serum glutamate levels as predictors.

Independent variables	OR	CI 95%	*p* value
Age	1.019	0.970–1.070	0.46
Gender	1.214	0.41–1.449	
Serum GLU 0–3	1.019	0.994–1.045	0.144
Serum GLU 5–7	1.029	1.005–1.055	**0.019**
Serum GLU 14	1.004	0.987–1.023	0.626

OR, odds ratio; CI, confidence interval; CSF, cerebrospinal fluid; GLU, glutamate.

Significant values are in [bold].

No association was found between CSF and serum aspartate levels and ICP, lesions in the CT scan, GOS, and the presence of neurological deficits (data not shown).

## Discussion

In this prospective study consisting of 45 participants with SAH with inserted an external ventricular CSF drainage, glutamate, aspartate levels in serum, and GOT1 activity, glutamate and aspartate levels in CSF were found to be constantly and significantly elevated. Moreover, high serum glutamate levels at days 5–7 post-SAH were significantly higher in patients with poor neurological status at admission. Importantly, we found that serum glutamate level and CSF GOT1 activity during the first week after SAH were significantly higher in patients with the presence of cerebral ischemia on later CT scan compared to those without cerebral ischemia, and were independent predictors of elevated ICP. Furthermore, serum glutamate levels and CSF GOT1 activity in the first three days after SAH were significantly higher in patients with longer hospitalizations, with the presence of severe neurological deficits at discharge, and with poor outcomes 3 months after SAH.

Neurological examination and brain imaging are currently the only reliable method for evaluating the severity of neurological damage in SAH patients^32,33^. To the best of our knowledge, this is the first study indicating serum glutamate monitoring at admission as a potential tool for the evaluation of neurological damage in the first days after SAH. Glutamate excitotoxicity is one of the most important mechanisms involved in early brain injury after neurotrauma^34,35^. It has been reported that extracellular glutamate levels rise within the first few minutes after SAH and peak at 40 min, then remain elevated for days^36,37^. In a rat model of SAH, the prolonged duration of depolarization and high extracellular glutamate levels were the only two factors that worsened the neuronal injury^38^. Moreover, the blood–brain glutamate balance plays an important role in both peripheral organs and CNS after neurotrauma, including SAH^14,19,39^. Impaired BBB function can allow an influx of glutamate from the blood to the brain, causing subsequent neuronal damage, as previously demonstrated in animal studies^40^. The blood-to-brain glutamate efflux and vice versa might contribute to the reported correlation between high serum glutamate levels and poor patient neurological status at admission, followed by poor outcome after SAH. These findings support the hypothesis that the blood–brain glutamate balance plays an essential role in the neuropathology of neurotrauma ^40–42^.

Previously, elevated serum glutamate and GOT1 levels were reported to be correlated with poor outcomes in ischemic stroke patients^43^. Similarly, in our study the levels of glutamate in the serum and CSF GOT1 activity correlated with the presence of a neurological deficit at discharge and with a poor outcome 3 months later. Few studies reported positive correlations between extracellular brain glutamate levels and neurological outcome in SAH patients^44,45^. In our study, high CSF glutamate levels at 14 days only were correlated with worse GOS. This may be due to the relatively high variability in the brain glutamate levels and the small number of SAH participants in our study. In addition, our results show that the CSF GOT1 level is a more sensitive marker for the severity of brain damage and the neurological outcome 3 months later.

Notably, activity of GOT1 but not GPT were significantly elevated in SAH participants, supporting recent studies that reported the central role of GOT1 in glutamate metabolism ^43^. Moreover, CSF GOT1 activity and serum glutamate levels were found to be higher in patients with ICP and were independent predictors of elevated ICP. Cerebral edema formation, an independent risk factor for poor outcome after SAH, is thought to be a consequence of astrocyte swelling as a result of glutamate excitotoxicity^11,46^. This finding provides exciting new evidence for the possible involvement of GOT1 in early brain events and glutamate regulation in SAH. One can assume that upregulation of brain GOT1 expression and activity in SAH patients may be part of the brain's early neuroprotective mechanism in response to glutamate excitotoxicity.

Although levels of aspartate in the blood and CSF were also significantly high, no correlation was found with early neurological damage or neurological outcome at discharge and 3 months later. Aspartate is a metabolite of GOT1 transaminase activity converting glutamate to alpha-ketoglutarate and aspartate. As far as is known, aspartate does not have a direct neurotoxic effect like glutamate and its levels also depend on the transaminase activity of the enzymes. This could be one of the explanations why in our study aspartate was less sensitive in reflecting short-term/long-term neurological damage in SAH patients.

The reported alteration in peripheral and central glutamate homeostasis is clinically important not only as a potential diagnostic/prognostic tool, but also as a potential target for therapeutic intervention and for the development of new drugs^47,48^. Evidence for reduction in blood glutamate concentration after blood-glutamate scavengers administration has been shown to have a significant neuroprotective effect in the SAH and stroke animal model and for reduced mortality after ischemic stroke in patients^41,47^.

### Strengths and limitations

To the best of our knowledge, this is the first time that serum glutamate and CSF GOT1 levels were shown to be constantly elevated in SAH and associated with early neurological damage and poor clinical outcome.

This study has some limitations that should be discussed. First, since we selected a limited number of patients who needed insertion of an external ventricular drain for clinical purposes, our data had to be confirmed in a larger cohort of patients with different severity of the injury. Second, CSF and plasma glutamate levels were analyzed in two different control groups, which could have led to misinterpretation of the results; on the other hand, it should be noted that both CSF GOT1 and GPT enzymes were not elevated in the TNS control group.

## Conclusion

In patients with SAH, serum glutamate and CSF GOT1 appear to be potential markers for early neurological damage during hospitalization, and for poor outcomes 3 months later. GOT1 activity in SAH participants was significantly higher in patients with increased ICP and cerebral ischemia, indicating early enzyme involvement in the neuropathology of SAH patients. However, these are preliminary results that need to be verified in a larger group.

## Supplementary Information

Supplementary Tables.

## Data Availability

The data presented in this study are available on request from the corresponding author.
