# Supplementary material for: Substantially elevated serum glutamate and CSF GOT-1 levels associated with cerebral ischemia and poor neurological outcomes in subarachnoid hemorrhage patients
Source: Sci Rep. 2023 Mar 31;13:5246. doi: 10.1038/s41598-023-32302-3 (PMC10066256; doi:10.1038/s41598-023-32302-3)
Supplement: Supplementary file 1 — Supplementary Tables. [file 41598_2023_32302_MOESM1_ESM.doc]

# Supp. Table 1 Summary of 14 TNS control group

| **N** | **Gender** | **Age** | **Treatment** | **Histology** |
| --- | --- | --- | --- | --- |
| 1 | f | 66 | TNS | non functioning pituitary macroadenoma |
| 2 | f | 37 | TNS | ACTH-secreting pituitary adenoma |
| 3 | m | 55 | TNS | non functioning pituitary macroadenoma |
| 4 | f | 25 | TNS | GH-secreting pituitary adenoma |
| 5 | f | 53 | TNS | non functioning pituitary macroadenoma |
| 6 | f | 43 | TNS | non functioning pituitary macroadenoma |
| 7 | f | 47 | TNS | non functioning pituitary macroadenoma |
| 8 | m | 61 | TNS | non functioning pituitary macroadenoma  recurrence |
| 9 | m | 44 | TNS | non functioning pituitary macroadenoma |
| 10 | f | 67 | TNS | non functioning pituitary macroadenoma |
| 11 | f | 44 | TNS | PRL-secreting pituitary adenoma |
| 12 | f | 54 | TNS | non functioning pituitary macroadenoma |
| 13 | m | 51 | TNS | non functioning pituitary macroadenoma |
| 14 | m | 39 | TNS | non functioning pituitary macroadenoma |

M: male; F: female; TNS: transsphenoidal; PRL: prolactin.

# Supp. Table 2 Summary of 15 healthy control group

| **No.** | **Gender** | **Age** |
| --- | --- | --- |
| 1 | f | 41 |
| 2 | f | 45 |
| 3 | f | 49 |
| 4 | m | 62 |
| 5 | m | 48 |
| 6 | m | 54 |
| 7 | f | 67 |
| 8 | f | 58 |
| 9 | f | 58 |
| 10 | f | 54 |
| 11 | f | 53 |
| 12 | m | 54 |
| 13 | f | 53 |
| 14 | f | 54 |
| 15 | m | 64 |

M: male; F: female.
